# SARS-CoV-2 screening of asymptomatic healthcare workers

**DOI:** 10.1017/ice.2020.361

**Published:** 2020-07-23

**Authors:** Andrew P. Jameson, Matthew P. Biersack, Tara M. Sebastian, Liberty R. Jacques

**Affiliations:** 1 Michigan State University College of Human Medicine, Grand Rapids, Michigan; 2 Mercy Health Saint Mary’s Hospital, Grand Rapids, Michigan

The emergence of the novel severe acute respiratory syndrome coronavirus 2 (SARS-CoV-2) and its associated coronavirus disease (COVID-19) has evolved into a global pandemic that, as of June 9, 2020, has taken >400,000 lives worldwide and has halted public life.^1^

Many reports have now established that asymptomatic and presymptomatic individuals play an essential role in perpetuating the spread of disease.^1–4^ Transmission rates within the healthcare setting have varied in the literature.^5–8^ We developed this protocol to determine the SARS-CoV-2 positivity rate among asymptomatic HCWs at our institution.

To effectively cohort patients, we developed a broad plan for screening asymptomatic and presymptomatic admissions in a 283-bed teaching hospital in an urban setting within Michigan. All inpatients admitted to the hospital were screened utilizing the GeneXpert RT-PCR platform (Cepheid, Sunnyvale, CA) via nasopharyngeal swabs. This screening program revealed that 1 in ~28 asymptomatic patients were positive for SARS-CoV-2.

At the time of HCW testing, the community burden of SARS-CoV-2 remained high, with 515 active cases per 100,000 county residents.^10^ Within the hospital itself, there were, on average, 3.8 new COVID-19 admissions per day in the 2 weeks preceding and the 2 weeks during the employee testing window.

## Methods

A voluntary SARS-CoV-2 testing program was offered to HCWs over a 2-week testing window. HCWs were excluded if they had symptoms of COVID-19 or previously tested positive for SARS-CoV-2. The program was made available to personnel who cared for COVID-19–positive patients in the ED or on the COVID-19 care unit. Screening was performed using nasopharyngeal swabs and the Cepheid GeneXpert RT-PCR assay. Staff were permitted to return to work while awaiting test results.

## Results

In total, 499 staff members were eligible for screening. Among them, 121 personnel volunteered to undergo testing (24.2% of those eligible). The results of all 121 tests were negative for SARS-CoV-2. Breaking down the uptake in testing by role: 6 of 53 of eligible respiratory therapists (11.3%) were tested, 33 of 92 eligible providers (35.9%) were tested, 71 of 262 eligible registered nurses (27.1%) were tested, and 11 of 82 of the eligible patient care assistants (13.4%) were tested.

## Discussion

The voluntary hospital staff testing program described here was implemented as a method of ensuring the safety of our personnel and patients from the established threat of asymptomatic transmission. Had any staff members received a positive test result, appropriate isolation measures would have been implemented to prevent viral spread, including a 10-day minimum administrative leave. The negative results of all tested individuals allowed these personnel to return to work in confidence and also informed the hospital’s decision to not continue routine testing of employees.

The 0% positive test rate among asymptomatic staff, despite the local community and hospital system experiencing a large burden of COVID-19 cases, is a testament to the ongoing work underway to ensure safety throughout the hospital. The following precautionary measures were implemented at our hospital:

- Universal SARS-CoV-2 testing of all patients admitted to the hospital, regardless of symptomatology or reason for stay
- Testing of all patients undergoing surgical procedures 24–48 hours prior to operation
- Isolation of all positive patients into designated COVID-19 care units
- Negative pressure ventilation systems for all COVID-19 care floors
- Personal protective equipment requirements including surgical masks and universal precautions on all floors, with the addition of gowns and eye protection on COVID-19 units
- Mandatory N95 mask or PAPR/CAPR use for any aerosol-generating procedures in COVID-19 units
- “No visitors” policy throughout the hospital, absent exigent circumstances (in accordance with Michigan’s March 14 executive order)
- Universal symptom screening of all staff arriving to work, excluding workers if they presented with any of the following symptoms: fever, cough, shortness of breath, chills, body aches, loss of taste, or loss of smell.

This testing was arranged through a COVID-specific Colleague Health Hotline designated to have a very low threshold for testing.

Adherence to this protocol has been of utmost priority throughout the hospital, in part because the ramifications of nosocomial transmission became evident early in the pandemic. The hospital had numerous instances of SARS-CoV-2–positive patients admitted to non–COVID-19 units, with a significant delay in diagnosis due to atypical clinical presentations. This repeated exposure to SARS-CoV-2–positive patients on non–COVID-19 units informed the decision to proceed with this protocol.

The uptake in testing among potentially exposed healthcare workers was also measured. Moreover, the 24.2% of eligible healthcare workers pursuing testing was lower than we expected. It is unclear whether this represents reluctance to undergo a diagnostic nasopharyngeal swab or confidence in the organizational approach. Regardless, this relatively low uptake does not support routine testing as an effective method to improve workforce confidence or safety.

In the months since implementation, adherence to the listed protective measures has been central to the safety of the hospital community and has contributed to the lack of positive testing among asymptomatic HCWs. As statewide regulations and social distancing restrictions begin to relax, it is essential to adequately protect our healthcare workforce. The infection control methods described have demonstrated how this organization was able to effectively protect this vital resource. Extensive testing of employees does not seem to be cost-effective or necessary when strong symptom screening and infection control policies are in place. As hospitals and communities prepare for the next phase of the pandemic, we recommend close monitoring of employee symptoms, rapid access to testing when symptoms develop, strong infection control practices, and broad testing of patients to effectively cohort patients as an alternative to testing asymptomatic employees.
